# Legibility: knowing disability in medical education inclusion

**DOI:** 10.1007/s10459-023-10268-1

**Published:** 2023-07-21

**Authors:** Neera R. Jain

**Affiliations:** Centre for Medical and Health Sciences Education, Waipapa Taumata Rau – University of Auckland School of Medicine, Private Bag 92019, Auckland, 1142 New Zealand

**Keywords:** Disability experiences, Disability inclusion, Critical disability studies, Equity, diversity, and inclusion, Medical education, Student perspectives, United States

## Abstract

How medical students, their teachers, and school administrators understand disability appears connected to ongoing, unequal access to medical education for disabled people. The stigmatization of disability within medical education affects students’ disability disclosures, yet few studies have explored how understandings of disability influence inclusion practices beyond individual student actions. This paper develops the concept of *legibility*, derived from a constructivist grounded theory study that examined disability inclusion at four U.S. medical schools through interviews with 19 disabled students and 27 school officials (faculty and administrators). With two dimensions (recognition and assessment of possibility), legibility demonstrates that knowing disability is relational, contextual, and equivocal. Drawing from the field of disability studies, the paper argues that the current paradigm of disability inclusion demands that students’ disability experiences be highly legible to themselves and others, yet increased legibility comes with potential risk due to prevalent ableism. While individual interactions can shift understandings of disability towards greater inclusivity, systemic action that embeds liberating discourses of disability into medical education is needed.

**Supplementary Information:**

The online version contains supplementary material available at 10.1007/s10459-023-10268-1.

## Introduction

Equitable access to medical education remains elusive for disabled people.^1^ Despite increasing international attention to this problem (Meeks et al., 2020b), disabled learners and physicians report significant barriers to access in the structure and culture of medical education, including labyrinthine policy and practice (Jarus et al., 2022; Petersen et al., 2022), insufficient accommodation (Meeks & Jain, 2018), and hostile work environments (Meeks et al., 2022). How medical students, their teachers, and school administrators understand disability appears to play a significant role in this ongoing inequity. The stigmatization of disability within medical education impedes disclosure of disability and accommodation requests (BMA, 2020; Bulk et al., 2017; Meeks et al., 2020c). Policies that construct disability as a threat to medicine and incompatible with the physician role also constrain access (McKee et al., 2016; Shrewsbury et al., 2018; Zazove et al., 2016). Despite the importance of how conceptualizations of disability impact inclusion, few studies have explored this matter in depth beyond its effects on individual disabled student actions (Bulk et al., 2017; Easterbrook et al., 2015). Disability studies theories suggest that conceptualizations of disability are relational and drive collective social action (Goodley, 2017; Kafer, 2013). This relational focus appears critical to the problem of inequitable access because the interactive process between disabled students and their schools to determine accommodations is central to inclusion (Laird et al., 2020). The interpersonal effects of knowing disability on inclusion practice remain undertheorized in medical education.

This paper develops the notion of *legibility*, which conceptualizes the political and relational dynamics that inform understandings of disability and action towards disability inclusion in medical education. Disability inclusion refers to processes by which barriers to participation are removed for disabled students. In line with its use in disability studies scholarship (Brilmyer & Lee, 2023; Evans, 2017; Hamraie, 2017; Nusbaum & Lester), I use the term legibility to suggest that how disability is understood conditions how it is recognized, and whether it will be regarded as a legitimate way of being internally, interpersonally, and institutionally. Previous scholars have discussed legibility related to individuals authoring their disability identity (Evans, 2017), how conceptualizations of humans inform design practice (Hamraie, 2017; Nusbaum & Lester, 2021), and how categorization systems can exclude or confer legitimacy (Brilmyer & Lee, 2023). These discussions highlight the politics of disability recognition: its potential to unlock access, legitimacy, community, and belonging, while raising the potential for surveillance and exclusion. My conceptualization of legibility focuses on how the individual and collective meanings made of disability affect access to medical education. Built through a constructivist grounded theory study of disability inclusion at four U.S. medical schools, legibility is discernable in the accounts of school officials (faculty and administrators) and disabled students. I begin by exploring disability studies theories that have sought to explain how conceptualizations of disability affect action. Then, I provide context by exploring how conceptualizations of disability have been discussed within disability inclusion research in medical education. After detailing methodology, I discuss the two dimensions of legibility, recognition and assessment of possibility, and their implications for practice. This analysis will surface the consequences of legibility under conditions of higher and lower levels of immediate recognition. Ultimately, I argue that the current paradigm of disability inclusion in medical education requires that learners’ disability experiences be highly legible to themselves and others. Yet, increased legibility comes with potential risk in a context saturated in ableism. Ableism refers to a system of beliefs and practices that uphold a hierarchy of bodies and minds (Campbell, 2009). The construct of legibility can inform interactions about disability that contest ableism and identify areas for systemic change.

### Theorizing disability

The field of disability studies debates the construct of disability and what meaning we make of it (Goodley, 2017; Shakespeare, 2013). Broadly speaking, the field questions realist accounts of disability, although such questioning can take distinct forms. Three theoretical positions—the medical, social, and political-relational models—provide distinct understandings of disability and each has implications for how disability is framed and treated. This debate is political. Models represent power struggles over what counts as normal, who decides, and how society ought to respond to difference. These models variously align with four common discourses of disability: individual inability, contextual production, valuable difference, and legal rights (Table 1). I will return to these discourses and models when I discuss legibility to explore their relevance to participant accounts and implications for legibility.

**Table 1:** Alignment between theoretical models of disability and disability discourses

		THEORETICAL MODELS OF DISABILITY		
		Medical	Social	Political-Relational
**DISABILITY DISCOURSES**	**Individual Inability**	Supports	Refutes	Refutes
	**Contextual Production**	Refutes	Supports	Supports
	**Valuable Difference**	Refutes	Ambiguous	Supports
	**Legal Rights**	Refutes	Supports	Transcends

Typifying a pure realist account of disability, the medical model assumes disability is an objective fact of the body. A diagnosis and the experiences that follow can be known and predicted by professionals through measurement and evaluation that place supposedly objective and bias-free norms onto bodyminds.^2^ Within this way of knowing disability, there is no meaningful distinction between impairment (the embodied experience) and disability (Oliver, 1996). Disability is something that is inherently defective and pathological within a person (Campbell, 2009). This state of being becomes a negative ontology, “a straightforward and obvious departure from normalcy” (Titchkosky, 2007, p. 105), where disabled bodyminds are understood by what they cannot do in reference to an assumed normal (Clare, 2017; Davis, 1995). An inherent quality of the body, disability is an individual problem that causes non-participation in society (Titchkosky, 2011). These understandings support discourses wherein disability is synonymous with *individual inability.* Because society is not directly implicated in individual problems, there is no duty for a collective response (Hahn, 1988). Individuals must seek care and rehabilitation to cure or minimize disability, and if this proves impossible, they must find ways to adjust to their condition (Campbell, 2009; Goodley, 2017). Any positive social action to include disabled people in society is considered benevolent, as disability is socially undesirable and therefore justifiably excluded (Schweik, 2009). Societal efforts are otherwise oriented towards medical and technological solutions to free people from disability (Clare, 2017; Mitchell & Snyder, 2015). The medical model does not support a discourse of disabled peoples’ *legal rights* to equal access. Paradoxically though, some scholars have argued that the Americans with Disabilities Act (ADA, 1990, 2008) aligns with the medical model of disability: dependence on a biomedical definition of disability in the ADA presupposes disability as individual inability (Donoghue, 2003; Skyer, 2019).

The social model is a constructivist theory of disability that contests the deficit-oriented formulations central to the medical model. The central idea of early disability rights movements (Shakespeare, 2013), the social model was initially formulated by activists in the UK, the Union of the Physically Impaired Against Segregation (UPIAS, 1975), and further developed by disabled scholars (Oliver, 1996). The model conceives of disability as oppression created by social barriers. In this framework, people have impairments but society disables them through discriminatory practices that create physical inaccessibility, social isolation, economic dependence, and removal of autonomy (Oliver, 1996). Thus, impairments are not theorized as socially constructed, but treated as neutral. Instead, the focus is on how disability is generated by societal arrangements, supporting a discourse of disability as *contextually produced*. By shifting the focus to societal arrangements, the social model becomes a political tool that fosters organizing around a collective identity (Garland-Thomson, 1997; Linton, 1998; Oliver, 1996). By problematizing societal arrangements rather than individual embodiments, the social model promises disabled peoples’ liberation, requiring societies to identify and remove barriers to participation (Oliver, 1996; Shakespeare, 2013). This framing supports a discourse of *legal rights*, necessitating policy development to fuel architectural changes, the provision of accommodations (adjustments to policy and practice), and equivalent access to social and economic activities (Scotch, 2000). The theory has underpinned disability rights movements across the world (Charlton, 1998; Driedger, 1989), with its influence discernable in various public policy developments (Scotch, 2000, 2001), such as the ADA (1990) and the United Nations Convention on the Rights of Persons with Disabilities (2006).

The political-relational model of disability, advocated by Kafer (2013), builds upon the social model and reforms it using critiques from feminist, queer, and ableism scholarship (Campbell, 2009; McRuer, 2006; Wendell, 1989). While the medical model presumes the existence of a normal and natural body, which disability represents deviation from, the political-relational model asserts that ideas of normalcy and deviance are themselves constructed phenomena (Campbell, 2009; Davis, 1995). The problem of disability moves from the individual to “inaccessible buildings, discriminatory attitudes, and ideological systems that attribute normalcy and deviance to particular minds and bodies” (Kafer, 2013, p. 6). Informed by feminist critiques of the social model, Kafer (2013) recognizes that embodied (impairment) experiences are part of disability, while understanding that these experiences are socially mediated. This framing holds ambiguity: experience cannot be discounted, but it is open to interpretation nonetheless. In her work, Kafer (2013, p. 4) notes that she is disabled but “is not interested in becoming more disabled.” Yet, she argues that disability is not something that ought to be expunged. Disability experiences can bring joy and value to a person’s life. And, how disability is experienced does not automatically follow from the “fact” of disability. In this framework, disability is best understood as relational, “experienced in and through relationships” (Kafer, 2013, p. 8) with human and non-human others; an assemblage of understandings and encounters with individuals, environments, and governing processes. As disability is fluid, shifting over time and space, this theory supports an understanding of disability as *contextually produced*. Disability is also political, inevitably entangled with power relations, and thus “can be critiqued, contested, and transformed” (Kafer, 2013, p. 9). A political-relational understanding, then, invites excavation and critique of the social relations that shape disabled people’s experiences. Through such a critique of power relations, the political-relational model operates in line with anti-ableism and crip theory to call for societal change that not only seeks to include disabled people into society as it currently exists but to transform society in ways that fundamentally value all forms of humanity (Campbell, 2009; McRuer, 2006). As such, this model opens the potential for a discourse of disability as *valuable difference*, a form of humanity that brings unique assets. While the political-relational model supports disabled peoples’ rights to access, the call for transformation moves beyond a *legal rights* discourse. In relation to legibility, the political-relational model of disability invites exploration of the interpersonal dimensions important to participant understandings of disability and resultant actions.

### Knowing disability in medical education

Disability disclosure and accommodations have been positioned as critical components of disability inclusion in medical education. Both are shaped by the meanings that disabled learners and others in the environment—peers, teachers, administrators—append to disability. Several studies have demonstrated that, in medicine, individuals with a condition that legally qualifies as a disability do not always self-identify as disabled (Jerome et al., 2022; Miller et al., 2009). Similarly, a recent British Medical Association (2020) survey of disabled doctors and medical students found that respondents remained uncertain about what qualifies as disability. This suggests some people who may benefit from accommodations do not pursue them due to how disability is understood. Identifying oneself as disabled appears further complicated insofar as disability is stigmatized in the field, aligned with notions of incompetence and vulnerability, and therefore perceived incompatible with a medical learner role (Fox et al., 2011; Meeks & Jain, 2018; Meeks et al., 2020c; Murphy et al., 2022; Nolan et al., 2015; Stergiopoulos et al., 2018). Studies document that learners strategically disclose and cover disability experiences in response to the potential for stigmatization, seeking to elevate perceptions of their competence (Easterbrook et al., 2015; Mayer et al., 2022; Sibbald and Beagan, 2022). However, discussions of disclosure have tended to focus on learners’ perceptions of others’ attitudes in the field and the impact this has on learner behavior alone, without excavating its impact within a relational process of inclusion. Further, such work has focused on the negative effects of stigmatizing discourses (e.g., marginalization, Bulk et al., 2017) but has not yet explored enabling relational discourses and processes. Finally, by treating institutional disclosure as a singular act that unlocks access to education (e.g., in studies of prevalence), existing studies have left unexplored how ongoing disclosures of disability experiences (e.g., discussions of barriers encountered) unfold and influence educational access. By framing disclosure as a singular act, rather than a process of uncovering layers of lived experience (Evans, 2017), such discussions have largely focused on experiences of learners with less readily apparent disabilities whose disability status is not disclosed by default. This omission obscures the complexity of disclosure for learners with more readily-apparent disabilities.

How key players conceptualize disability is important given that U.S. law (i.e., the ADA) requires a robust *interactive process* to determine appropriate accommodations for educational settings (Laird et al., 2020). This process relies on an in-depth exploration of potential accommodations between a responsible official, program faculty and staff, and the student (Laird et al., 2020). Scholars argue that this process necessitates at least one school official who is (a) knowledgeable about best and emerging practices for accommodation in medical education, (b) familiar with associated legal requirements, and (c) not in a role wherein they will assess the learner (Babbitt & Lee, 2016; Meeks & Jain, 2018; Meeks et al., 2019b, 2021). Bulk et al. (2019) further identified two dimensions of the disability experience—its “visibility” and its “onset within the life course”—that affected how healthcare clinicians and learners approached self-advocacy and accommodations, contending that these dimensions should inform a nuanced interactive process. Yet, their research did not theorize how the interactive process currently operates. Several case studies have documented successful, complex accommodation delivery in clinical settings (Jauregui et al., 2020; Meeks et al., 2015, 2018). These studies demonstrate how accommodations were made, showing aspects of the interactive process to implement accommodations. However, these pieces focus on successes and do not excavate the understandings that enabled or threatened these outcomes. While some studies have begun to examine aspects of the interactive process, the dynamics of disability understandings between students, faculty, and administrators and their influence on practice have not yet been sufficiently unearthed through research.

## Methodology

This paper presents a theoretical concept, legibility, developed through a constructivist grounded theory (CGT) project that explored how disability inclusion was enacted at four U.S. medical schools (Jain, 2020b). CGT uses inductive and iterative techniques to develop theory that explains social processes (Charmaz, 2014). Underpinned by a relativist ontology and subjectivist epistemology, CGT recognizes that knowledge is partial and contextual, informed by a researcher’s positionality and values, with data open to interpretation (Mills et al., 2006; Charmaz, 2014). This analysis includes interviews with 27 school officials and 19 disabled students.

After receiving university ethics approval, institutions were recruited that demonstrated potential to exceed a compliance approach to disability inclusion (Jain, 2020b). To do so, I identified schools with signs of institutional disability champions and other potential indicators of a positive disability culture (e.g., affiliated faculty publishing research regarding disability inclusion in medical education or disability curriculum initiatives, publicly identified disabled faculty, dedicated disability resource professional for medical school, disabled medical students’ group, and technical standards stating the possibility for accommodations). For an in-depth discussion of this selection process, see Jain, 2020a, pp. 50–51. I contacted leadership at 20 schools via unsolicited emails and third-party introductions to gain institutional consent to recruit faculty, staff, and students. Four Deans consented to institutional participation with agreement that schools would not be named and leadership would not be informed who participated. Additional institutional ethics approval was required at two schools. The four medical schools are located at public universities in Southern (one), Central (two), and Western (one) US.

A school leader emailed recruitment invitations to all undergraduate medical students and faculty. Students with self-identified disabilities were invited to participate and network sampling aided recruitment. School officials with roles important to disability inclusion were invited to participate through direct emails, identified through public information and interviews. Most recruitment materials did not define disability, however, the student recruitment email specified, “All experiences of disability (learning, psychological, physical, sensory, chronic health, and AD/HD) will be included in this research.” Interested individuals contacted the researcher and provided informed consent. Nineteen students and 27 school officials participated across the four schools, totaling 46 participants. Students represented a spread of disability experiences (physical, cognitive, mental health, chronic health) and years in medical school (first through fourth year). School officials represented all levels of faculty and administration (program coordinator through vice dean). I provide only high-level details about participants to limit identifiability of schools and individuals within a small community, given the significant stigmatization of disability and perceived risk of sharing experiences in medicine (Jarus et al., 2022; Meeks & Jain, 2018).

### Strong reflexivity

I am an Indian-American, biracialized, cis woman who, at this moment, identifies as non-disabled and is committed to justice in research and health science education. I previously led disability services for health science students at two U.S. universities. These positions drove my engagement in this work, shaping “what [I] can see” (Charmaz, 2014, p. 27). As the primary actor developing this research, methodological self-consciousness (Charmaz, 2020) and strong reflexivity (Harding, 1991) were necessary. Methodological self-consciousness requires “a deeply reflexive gaze on how our perspectives, privileges, and priorities affect our data, actions, and nascent analyses” (Charmaz, 2020, p. 3). Charmaz (2017, p. 36) advocates that this be achieved through strong reflexivity: multi-faceted and continuous reflection on our social location, imagining how participants see the researcher and the research project from their standpoints, viewing the research within the larger social and cultural context, and using this information to shape research processes. I critically reflected on myself, the data, extant theory, and the research process throughout. While shaping the research project, generating, and analyzing data, I critically examined my practice and thinking through memos, discussions with colleagues, reading disability studies theory and activist writings, and in conversations with disabled activists, medical students, and colleagues. I questioned how my actions and interpretations were shaped by my lived experience and challenged myself to resist rote explanations, using these reflections to shift my practice (Charmaz, 2020; Harding, 1991). CGT techniques facilitated this work: treating all data as problematic, staying close to the data, surfacing tacit meaning in interviews, constant comparison, seeking new data, and incorporating extant theory (Charmaz, 2014). This strong reflexivity shaped the research and brought me to a new understanding of the constructed concept of disability and relationality within inclusion work. I began the research grounded in the legal definition of disability in the ADA and allied to the social model of disability. Through this research, I have come to understand disability in line with the political-relational model.

### Data generation and analysis

Data generation and analysis were iterative, with analysis commencing early to focus later stage activities. Following the heuristics of CGT (Charmaz, 2014), analysis comprised a non-linear process of memoing and coding that used the constant comparative method. These activities informed theoretical sampling to saturate categories and develop theoretical concepts towards theory-building.

Semi-structured interviews (45–180 min) were conducted by video conference or in person and explored participant experiences with disability inclusion. Initial interviews occurred between May and October 2017 (see Appendices A-C for semi-structured interview guides). To facilitate accessible interviews, I elicited access needs in advance and responded to participant needs for time to fully express themselves, energy levels, and desire to share their experiences through flexible interview length (Price & Kerschbaum, 2016). All interviews were transcribed and sent to participants for review, additions, and redactions. This process offered participants the opportunity to reconsider and clarify their statements. Several school officials made small edits to clarify statements and correct inaudible phrases.

Initial coding was line-by-line and inductive, attending to action and remaining close to the data. The constant comparative method was used to compare similarly coded data within and across interviews to develop focused codes (Charmaz, 2014). I also segmented and diagramed codes to compare processes, capture meanings and gaps to draw further connections. During analysis I looked at student and school official data separately and then together to identify similarities, differences, and interconnections among students, among school officials, and across the two groups. Memos and conceptual maps were used throughout analysis to capture developing concepts, divergent accounts, and tease out processes. Through these techniques, I identified areas to query in subsequent interviews. For example, after the meaning of disability and students’ relationship to the term appeared salient in initial interviews and correspondence with prospective participants, I added the question “can you tell me about your relationship to disability?” to subsequent student interviews and pursued the topic with school officials. These techniques advanced analysis towards more abstract codes and categories to facilitate theory development.

I used several forms of theoretical sampling, including abduction, re-analysis of existing data, extant literature, and seeking new data to achieve *theoretical saturation*, the conceptual density of repeating patterns in categories (Charmaz, 2014). Through abduction, theories of disability helped to elucidate the complex, divergent, and at times internally conflicted ways participants understood disability and interacted with inclusion processes. New data comprised six follow-up interviews conducted via video conference with three students and three school officials (August-October 2019). Participants were selected based on stated willingness to participate in additional conversations and potential to further theoretical development. Memos following each interview identified strategies to finalize the analysis. These interviews largely confirmed the developing analysis, providing additional examples of incidents that followed categories identified in earlier analyses without taking the theory in new directions.

Through the process of category development and subsequent theoretical sampling, I identified abstract theoretical concepts that subsumed focused codes and had “substantial analytical weight” (Charmaz, 2014, p. 247). These concepts were *legibility* (knowing disability) and *the capability imperative* (knowing medicine). These larger, abstract theoretical concepts provided interpretive frames to explicate, organize, and present the data. This paper presents one of these theoretical concepts, *legibility*, which conceptualizes how participants understood disability and how this informed participant actions related to inclusion (e.g., disability disclosure, requests for accommodation, the interactive process, accommodation determination process). In the following section I will discuss two dimensions of legibility: recognition and assessments of possibility.

## Legibility

### Recognition: self and other

For a student to disclose disability and make accommodation requests within the current paradigm of disability inclusion, they must recognize their experience as disability, realize that this status is connected to the right to ask for barrier-removal in the educational setting, and have others (including the institution) recognize this status in kind (see, e.g., Laird et al., 2020). Students participating in this study represented varied routes to recognition of their disability status. Some students entered medicine already recognizing their experience as disability. For those who did not come with a sense of recognition, some developed it through interactions with others within and outside medical school. Others were uncertain of whether to seek formal recognition of their status and request accommodations. School officials also had varying understandings of disability. While recognition may appear straightforward, connecting one’s experience to disability and associated rights is complicated by the divergent discourses of disability that circulate within a social context and their relative power. Four competing discourses of disability underpinned participant accounts and these influenced recognition. As discussed in the “theorizing disability” section above, these four discourses were individual inability, contextual production, valuable difference, and legal rights.

Strongly aligning with the medical model of disability, the *individual inability* discourse dominated participant accounts. Disability was understood as all-consuming, resulting in complete impairment and spreading burden to others. This was understandably at odds with students’ lived experiences. As a result, some students felt they could not or would not claim disability:

*I just think when you put disability next to your name, people are already thinking you can do less than other people, you know? It’s automatic. That is what the word is, dis-ability, you don’t have the ability to do certain things and thus, you can probably do less. (Student, 44)*

Many students, regardless of how readily-apparent their disability was to others, grappled with their simultaneous internalization and rejection of the inability discourse. The saturation of the inability discourse in wider culture, historical experiences, and in their medical school experiences reinforced its legitimacy, causing students to expect its acceptance and act accordingly. School officials also grappled with this discourse, with those positioning themselves as advocates for disability inclusion largely rejecting it, while acknowledging its dominance among their colleagues’ thinking.

Aligning with the social and political-relational models of disability, participant understandings of disability as *contextually produced* jostled against the individual inability discourse. The contextual production discourse emerged when students and school officials identified environmental barriers that created difficulty, rather than impairment itself.

*With ADHD, it’s not like I can’t, I just need tools in place to be able. And also, the only reason why I need all those tools is because everything is based on these [pedagogical] models that we already have proven are not great for anybody. 1% of people really learn from lecture and nothing else, if that. (Student, 30)*

However, recognizing the ways sociocultural arrangements produce disability at times hampered recognition. Students who recognized their experiences as contextually produced questioned whether they were “really” disabled, due to its association with individual inability. Yet, the discourse of contextual production is present in the logic of accommodations that offer a route to shift the context for individuals.

Some participants conceptualized disability as *valuable difference*, unique ways of thinking and being in the world that would benefit learning and medical practice. Participants characterized disability as valuable in terms of disability epistemology and its effects on patient care, near-peer learning, grit, and potential for innovation. This positive conceptualization of disability represented an *oppositional* or *outlaw ontology* (Campbell, 2001; Wrigley, 1997), a way of being that participants recognized as outside the dominant discourse of disability. For students, this understanding was often privately held and animated dissatisfaction with predominant negative conceptions of disability and institutional conditions. Disabled school officials were most likely to invoke the conception of valuable difference. Officials without lived experience usually developed this perspective working alongside disabled students or colleagues:

*It became clear to me when we’d have a child with a spinal cord injury how much they identified with [my disabled colleague] and not me. I remember one little boy… they got into an animated conversation and it was something really quite magical, because this little boy is going to have an image of a man who is a doctor, and his spinal cord injury has not prevented him from being able to consider still using his brain. (School Official, 31)*

Despite individuals reading disability as valuable, such a conception was rarely codified within institutional frameworks, with only one participating school activating disability in institutional diversity efforts. This erasure of disability as valuable difference on an institutional level empowered the inability discourse. However, belief in the valuable difference discourse could support recognition, with students describing this as a driver of political disclosure (Jain, 2020c) that invited peers to be open about their disabilities and access support services.

The legal rights discourse was an “absent-present.” The definition of disability contained in the Americans with Disabilities Act, Amendments Act (ADAA, 2008) prescribes who is disabled for legal purposes, and therefore eligible for educational accommodations. Given that the ADAA definition and associated regulations underpin and often drive disability inclusion practice in U.S. higher education (Meeks et al., 2020d), it could be expected that this discourse would be a central operating framework in the educational environment. However, the ADAA definition was not widely understood outside of those school officials directly responsible for determining accommodations. Several participating students, for example, did not recognize that their diagnoses “counted” as a disability under the law, reflecting previous findings (BMA, 2020; Jerome et al., 2022; Miller et al., 2009). Even those accessing accommodations questioned whether they were “really” disabled. The broadly conceived ADAA definition was distinct from participants’ narrow, colloquial understandings, which generated uncertainty about who counts as disabled and is eligible for accommodations. While most participants were aware that there was a legal basis to the provision of accommodations, the legislative requirements for schools, rights afforded students, and the ADAA’s reach beyond education was not well-understood.

The dominant discourse of disability—individual inability—conflicted with the other three modes of understanding—contextual, valuable, and legal. Participants developed these latter ways of knowing disability through their lived experiences and interactions. Through these understandings, new possibilities for self-knowledge and inclusion emerged. The overarching dominance of the individual inability discourse, however, tempered the possibilities presented by alternate discourses. The discourse of inability hampered recognition because of its stigmatizing power and because students did not relate with its implication that disabled people are profoundly incapable. The inability discourse, therefore, represented a gravitational force for participants to manage in the pursuit of inclusion. Alternative ways of knowing disability, especially when shared, fueled students’ ability to persist against the inability discourse. For school officials, alternative discourses operated as a resource to contest exclusion.

### Implications of recognition

The ways participants recognized disability had implications for students and inclusion practice. Participants held colloquial understandings of disability that characterized certain embodiments as paradigmatic of disability: use of a wheelchair, being blind or deaf, using visible assistive devices (e.g., cane, walker), and so on. Such embodiments were met with the greatest recognition, which participants often referred to as “real” disabilities. This recognition came with a sense of hypervisibility that held the greatest burden of the individual inability discourse. Yet, high recognition also came with a stronger application of the legal rights discourse, their eligibility for accommodations was unquestionable. For students whose experiences were not attributed “realness” (e.g., those experiences categorized as cognitive, psychological, or chronic health disabilities), recognition was not as immediate. This protected them from immediate application of the individual inability discourse, yet lower recognition also made connecting their experience to a legal rights discourse more challenging.

Experiences of disability with the highest recognition were far less common than other forms of disability at the studied schools (cf. Meeks et al., 2019a). Thus, students in this category occupied a token status (Kanter, 1977) at their medical schools and in clinical spaces, often the only visibly disabled person. Their high visibility and novelty in the environment could result in a feeling of constant surveillance:

*If you have a white jacket you might just blend with the others. I can’t do that, and so if I come in 10 min late, “Oh that’s the person, oh that’s her, I remember her” and because I have a physical disability you are more likely to be remembered as having done that than somebody else who doesn’t look different, they might forget your face. With me, they are not going to forget and so you are under a microscope. (Student, 44)*

Being immediately memorable meant always needing to be “on” and exhibiting peak performance. Participating students with high-recognition disabilities felt unable to be average, let alone do poorly, as this would be remembered and possibly logged as an inability indicator. They had to work to overcome or subvert attribution of the dominant individual inability discourse, often succeeding through leveraging the valuable difference discourse.

Students whose disability experiences were not readily attributed “realness” had different challenges associated with recognition. As one student described:

*The only time they really advertise that there is such a thing as accommodations is [during] orientation. They have a slide that has… a little statement of ADA… they’re like, “Oh yeah if you have accommodations just make sure you email this person.” That’s as far as they go to tell people what ADA is. And to me, I didn’t really know what that was for. I thought it was for people that had difficulties with, I don’t know, something very debilitating, for example if they’re in a wheelchair they would need certain accommodations for that, making sure there’s ramps or something. I never really thought about it as depression and anxiety being a disability. (Student, 8)*

This student’s account links the inability discourse with the concept of “real” disabilities. Together, these ideas led students to question their disability status and hesitate to claim the stigmatized label. The student also highlights the need for schools to demystify the legal rights discourse. Student accounts demonstrated that school officials often assumed disabled students already know what the ADA is, who is covered, and what accommodations might be possible. Thus, policies or communications that failed to clarify the breadth of coverage and possible responses moderated recognition, and therefore legibility of certain disability experiences. Unless students had used disability services in previous schooling, it was often not until they disclosed a diagnosis to a knowledgeable and trusted school official, peer, or other support person (e.g., a therapist) that their experience was recognizable to them as a disability, eligible for accommodations. This was apparent for several students who had not been referred to discuss possible accommodations upon disclosure of their diagnosis to a school official. These students interpreted the lack of referral to mean they were likely not eligible for accommodations or that no suitable accommodations existed when this may have reflected the school official’s limited understandings.

### Assessment of possibility

The second dimension of legibility, assessment of possibility, concerns foreseeing the barriers a student might encounter in medical education and practice, and assessing whether these barriers could be ameliorated. Many factors impacted the assessment of possibility. Not surprisingly perhaps, assessment was fundamentally wedded to recognition. Whereas highly-recognized disabilities opened up questions about barriers, less recognized disabilities did not always invite comparable concern. But, assessment hinged on other factors as well. Of importance were the lived experiences of participants, their observations and prior experiences with disability, and the knowledge and stories they had heard about disability experiences in medicine. Assessment was shaped by familiarity with medical education spaces, with students having less familiarity with required tasks and environments than school officials. Furthermore, legal discourses on disability refracted assessments of possibility. Not all students readily understood that accommodations were possible in medical school, even if they had used accommodations in previous education. Similarly, understanding the ADAA’s coverage of employment settings was critical to assessing future possibilities in medicine. Oftentimes, it was assumed that workplaces would not make accommodations and, as a result, whether accommodations should be provided in clinical education became a question. Given the different conceptions of disability in play, and the range of discourses surrounding disability, assessment could quite easily under or over-estimate barriers to participation, and therefore possibilities for change.

### Implications of assessing possibilities

With more immediate recognition came greater attention to systemic barrier-removal and anticipatory attention to individual accommodation needs. School officials conveyed greater confidence in their ability to identify accommodations for disabled students afforded high recognition (e.g., physical disabilities). This confidence fueled proactive attention to barriers and a collaborative approach to determining their removal. One school, for example, employed a team-based approach to identify accommodations and adjustments for students with apparent physical disabilities:

*We have a team of people that worked on accommodations for the first two years and so I think we all had on our radar like there were certain clerkship directors that I needed to talk to… I certainly didn’t feel solely responsible for directing that, I had a lot of support in that, and I also didn’t feel like I was being told. I knew that was something I needed to do… ahead of my surgery rotation, and I felt like I had the right support to say, “Who is the clerkship director for surgery and when do I need to meet with them?” (Student, 10)*

In this partnership approach, the group systematically reviewed technical skills required in clinical settings and developed adaptations, shadowing opportunities, and worked out any alternatives a student might need to participate in skill-building labs and clinical rotations. While incredibly valuable and representative of exemplary practice, there were some limitations. The group’s attention was focused on the physical tasks of clinical work, the most legible barriers students would encounter. The less obvious structural barriers, such as early start times and long clinical days, remained outside the anticipatory process. This is representative of a common issue among school officials. Their assessment of physical access concerns often overshadowed other structural barriers. From a student perspective, however, physical access was only one genre of barrier they experienced and, in many cases, the one they were most readily equipped to manage given their everyday adaptability.

Assessment of possibility had implications for medical school admissions, again tied to notions of recognition. While students who were afforded less immediate recognition reported fewer challenges related to admissions processes if they did not discuss their disability, all participating students with higher immediate recognition recounted stories of difficulty securing admission to medical school. For example, one student had an acceptance rescinded related to faculty concerns about accommodation possibilities, another was only admitted to one medical school. While school officials asserted that disability information is not used to exclude students from admission, some nonetheless described lingering uncertainties that students with “real” disabilities had the capability to become a physician. This often manifested as an imagined ceiling of inclusive possibilities in medicine, such as the following:

*I think it would be very difficult to have somebody who was blind in medical school… I cannot imagine how you could make it happen. You have to read psychology, you have to be able to look at slides… In fact, I think that that would truly be the only thing that at this point keep somebody out of the medical school environment, otherwise I think pretty much anything else is educable. (School Official, 11)*

While the specifics of the imagined ceiling varied, this phenomenon suggested the costs of recognition for assessments of possibility. In the statement above, there is recognition of visual impairment as a disability, which is combined with a limited assessment of possibility. Blindness is presumed incompatible with being a physician. Interestingly, blindness was suggested by multiple participants as the ceiling point, despite at least two physicians who successfully completed medical training while blind and practice psychiatry in the US (Hartman & Asbell, 1978; Smith, 2011). The imagined ceiling was often shaped by an individual’s assumptions about disability experiences and possible accommodations rather than deep knowledge of disability and inclusive possibilities.

Those school officials with less awareness of successful disabled physicians and learners, and less understanding of the legal parameters for accommodations, tended to make more limited assessments of possibility. However, even those who conveyed a general belief in possibility for disabled learners often described uncertainty about appropriate accommodations, especially for people whose disabilities were less readily apparent. School officials communicated discomfort anticipating the barriers these students might encounter in clinical settings, as well as a general lack of understanding about their diagnoses. Many school officials, despite awareness of the legal definition of disability and their medical training, did not understand learning disabilities, psychological diagnoses, and AD/HD, nor possible accommodations to remove barriers.

*It would be a lot easier if there were more people with common physical and sensory disabilities where… it was very clear what the student was capable of and was not, and you could have a focused discussion about why this was or wasn’t important to their training as a physician. It’s much harder to do when what you’re dealing with is a neuropsychology report on someone’s learning disability. It’s just harder.* (School Official, 6)

This is a notable concern given the high prevalence of students with these disability experiences in medical education (Meeks et al., 2019a). Without strong understanding of less-readily apparent disabilities, school officials struggled with barrier identification and removal. The illegibility of these experiences prompted several different responses. Some questioned the validity of students’ disability experiences and subsequently the necessity for accommodations. This created tension between faculty and disability experts, or students and faculty. Another group of school officials recognized their limited expertise and were satisfied to delegate responsibility to disability experts and trust their decision-making. While somewhat more favorable, this response sometimes resulted in faculty disengagement with access concerns. A third group represented school officials with responsibility for accommodation decision-making on committees, but who did not understand these disabilities or possible accommodations. This final group was particularly concerning. Without a knowledgeable student or school official to intervene on decision-making, poor service for students resulted. Some school officials were aware that students with less legible impairments were not as well served by their schools. Many, however, were less aware of their interpretive limitations, and simply did not anticipate that these students might require accommodations beyond exam settings.

When disability experiences were not legible to school officials, this heightened the burden for students to self-identify barriers, prove their access concerns, and identify solutions. Students often could not fully-anticipate barriers and remedies until they entered the environment. This meant students might not realize that they required accommodations until they were in the thick of intensive clinical experiences. Several students who found themselves in this situation described a painful, disruptive bureaucratic process to identify and implement accommodations that negatively affected their clinical performance. This reactive practice particularly disadvantaged students who were less confident self-advocates, with less trust in school administration, and who did not fully understand their legal rights. While schools have no legal obligation to anticipate the accommodations students need, responsive practice for those students with less-ready recognition was in stark contrast to the proactive, collaborative experiences described by students afforded ready recognition.

### Shifting legibility, shifting practice

Legibility was not static. Interactions between students and school officials, among students, and school officials could change how participants understood disability. These dynamic movements demonstrate potential ways to shift practice. Students generally believed strongly in their potential to become successful physicians. Through connections with disabled physicians or more senior learners, some developed understandings of possible accommodations and adjustments that solidified their certainty. For others, it was through peers that they recognized their experience of barriers as a disability that might open the potential for accommodations:

*I have a group of really great friends and a few of them have ADHD. As we were talking about it* [my experiences], *they were like, “Everything you’re saying sounds like you need to go to the doctor… maybe meds will really help you, maybe you need accommodations… you need to see if there’s something else [going on].”* (Student, 30)

Some school officials supported opportunities to learn from peers by connecting students, though this generally happened ad-hoc, not through an organized effort.

School officials shifted legibility by sharing prior experience supporting students, discussing upcoming tasks in training, and organizing opportunities to visit clinical spaces to help identify potential barriers. As described previously, this could occur through an organized process, but for many students it was less formal:

*We talked about other things that could come up in medical school …* [the disability resource professional] *was able to say, “hey in the future, you’re going to have to be able to test range of motion”* [knowing] *I won’t be able to lift people’s limbs and manipulate them, and just like talking about thinking ahead so that we could prepare in advance for changes coming up.* (Student, 33)

When school officials openly discussed potential barriers and accommodation ideas with students, this clarified possibility and opened space for students to reveal barriers more readily. However, this kind of discussion did not occur for all students. Such discussions shifted collective understandings of disability and the potential for barrier removal. The reciprocal process of clarification advanced assessments of possibility, and therefore legibility.

Those with responsibility for determining accommodations generally had a broader sense of the potential for various accommodations. They shared success stories and cautionary tales to educate their colleagues.

*Every time a school screws up, I announce it to the Assistant Dean, Associate Dean, I send out [the case]. I mean, I’ve got the law on my side. I’m not breaking the law [by pushing for accommodations]. I am abiding the law… I keep saying, [do] you want a $400,000 penalty? (School Official, 13)*

Such actions aimed to clarify the institution’s legal obligation to provide accommodations, warn colleagues about the consequences for not complying, and to elevate collective possibility for disabled students. Together, these efforts shifted legibility among school officials.

The power differential between students and their schools, and the dominance of the individual inability discourse, created a burden for students to shift school officials’ perspectives towards a positive assessment of possibility. This created a complex sense of responsibility among students whose disabilities were readily recognized. All these students anticipated that their performance would determine the possibility for other students deemed like them to enter medicine. That is, how others came to understand their disability and potential to succeed in medicine would shape the legibility of similar others as viable candidates.

*People with disabilities are only recently “allowed” to go to medical school, so you’re the “prototype.” If the prototype “doesn’t work” it’s not going to go into further development for subsequent groups of people. So that’s the pressure that I feel… You want to make sure that the next guinea pigs get their chance too.* (Student, 46)

Under these conditions, students with readily apparent disabilities felt significant pressure to appear capable and likeable to shift school officials towards a positive assessment of possibility. They saw this as necessary for their own success and for future students like them. This adds further dimension to findings from Jarus et al. (2022) that suggested disabled students hesitated to provide honest feedback about their experiences to maintain a positive relationship with their schools.

## Concluding discussion

**Fig. 1:**
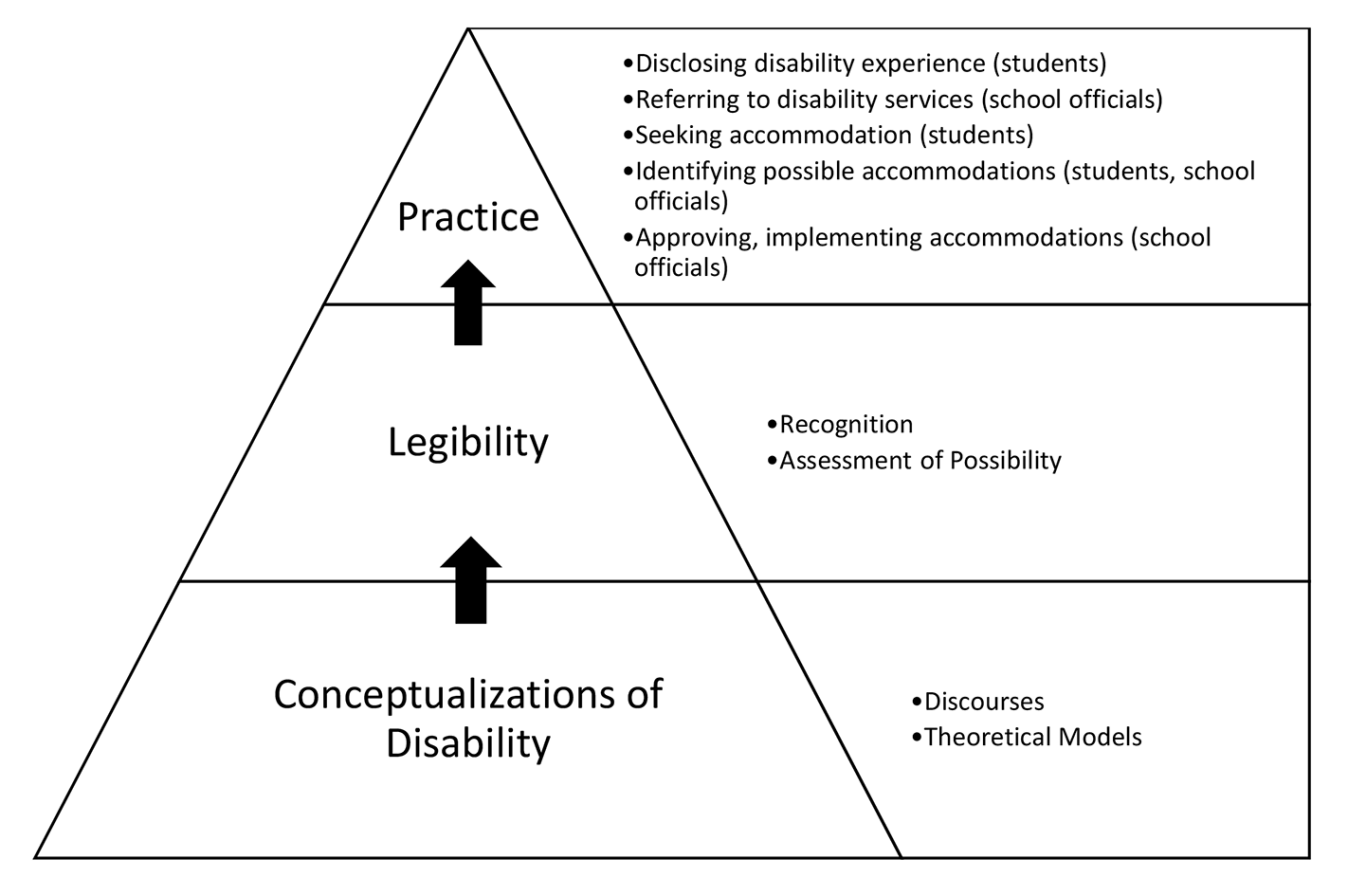
Legibility in Medical Education Inclusion

In the context of medical education, legibility is a spectrum of experiences contingent upon dimensions of recognition and assessments of possibility. Legibility is informed by conceptualizations of disability and, in turn, informs practice (see Fig. 1). This formulation encompasses what experiences and barriers were legible in medical education. Participating students and school officials were nestled amongst recognition and assessments of possibility in variable ways. Legibility was not static, nor consistent. Participant experiences suggest legibility will depend on one’s self-conception, operative concepts in a context, and other players’ readings of the disability experience. Legibility shifted through relationships, with potential for misrecognition and misunderstanding of disability experiences as well as the potential to develop greater clarity, resonance, and sense of possibility. However, any position on the spectrum of legibility was equivocal and contingent. Being recognized as disabled could be positive or negative, based on interpretations of disability and perceived possibilities for success in medicine. While greater recognition conferred legitimacy of one’s disability status, it did not guarantee a wholly positive experience in medical education. Students with more immediate recognition described beneficial and detrimental effects of this status, caught as they were between legitimacy and stigma. Those students whose experiences were less readily recognized as disability were more likely to have their legitimacy and rights questioned, and they experienced lower institutional confidence in determining accommodations. At the same time, their ability to “pass” as non-disabled afforded some freedom from stigmatizing judgements. Despite such variation, it was a constant that legibility had implications for inclusion; it impacted how students disclosed their disability experiences and how school officials responded to disclosure and facilitated accommodations. Ultimately, this concept explicates whether and how disabled people were legible *as successful medical students*.

Legibility suggests that how we know disability materializes (im)possibility for inclusion in medical education. The prevailing paradigm of disability inclusion in the U.S. and elsewhere is responsive. Medical schools do not have an affirmative duty to ensure access for disabled people. Instead, access is negotiated individually, upon request. Such a system hinges upon mutual recognition of disability status and belief that access is possible: high legibility is presupposed. Yet, within medical education, this demand for high legibility comes with risk, given the profession’s ableist conception of the physician as selfless superhuman (Jain, 2022), and the equation of disability with individual inability. These forces collectively suggest disability is a potentially disqualifying characteristic for medical students and a systemic burden. Through the accounts of disabled medical students and school officials, legibility demonstrates the power of interpersonal interactions to overcome or reinforce dominant discourses of inability, to find spaces of inclusivity or to reinforce exclusivity. Legibility can shift through relations. As a result, inclusion is incredibly contingent on people and the conceptual frameworks they come to adopt: the knowledge and beliefs about disability they carry, and their ability to contest the medical model of disability. These interpersonal relations are consequential, shaping how a student’s disability is recognized within the institution, and what access possibilities are created by school officials. These findings suggest interpersonal relations may be a space for intervention to improve inclusion experiences. Legibility also illuminates why individual student experiences of disability inclusion are inconsistent within and across institutions.

The political-relational model of disability is activated through the construct of legibility. Kafer (2013) contends that disability is created in relationship with human and non-human others. Disability is not reducible to a fact of the body. Disclosure and the interactive process, a discussion between people to determine disability and accommodations, ensure that disability experiences in medical and other higher-education contexts are relational. Yet, the power dynamics of these relations are important. Students are placed in a less powerful position due to the hierarchical context of education and the structure of disability disclosure and accommodation determination. Students must prove their disability and access needs against academic requirements (Laird et al., 2020). In participant accounts, disability remained tied to a discourse of individual inability with a limited discourse of legal rights. Thus, the politics of legibility are significant: the interactive process by which educational accommodations are determined is a political site that deserves critique. Legibility demonstrates that the multiple ways that learners and school officials conceptualized disability impacted their interactions with disability inclusion mechanisms. The limited ways that school officials conceptualized disability, recognized barriers, and subsequently acted is cause for concern. A political-relational lens suggests that the ways students and school officials interacted are not inevitable. Rather, they are the product of powerful ideas about disability and inclusion that require ongoing examination. Considering medicine’s professed desire to diversify the profession (Cohen et al., 2002; Murphy, 2021; Nivet, 2015; Roberts et al., 2014), the element of chance—who one interacts with and what ideas they carry about disability—seems insufficient to achieve just inclusion.

Discourses of disability played a significant part in legibility and illuminated the various models of disability active in medical school environments. While the scholarly analysis of disability tends to assume a consistent theory of disability (Goodley, 2017), in this study participants held multiple, sometimes conflicting, conceptualizations of disability. The co-existence of various conceptualizations contributed to misrecognition among learners and raised questions about the limits of access. Perhaps the presence of a range of disability discourses, including enabling discourses of contextual production, legal rights, and value, are representative of schools in transition away from the medical model of disability and its harmful implications for inclusion. However, the predominance of the individual inability discourse made knowing disability in alternative ways, and therefore the work of inclusion, a constant act of resistance. The medical model of disability still dominated these medical education spaces. The discourse of valuable difference was insufficiently present, often held privately by individuals and not reinforced institutionally. However, when active, this discourse operated as a tool for change.

The findings from this study showed that shifting legibility was possible on an individual level, requiring sharing among peers, between students and school officials, and between school officials. Individuals sharing their experiences and asserting value is powerful but can burden disabled people with the responsibility and risk for shifting the culture (Jain, 2020c). To empower resistant discourses, medical schools would need to attend to the ways policy, practice, and curriculum (formal and informal) construct disability. For example, when disability is framed as a risk to medicine (Shrewsbury et al., 2018), this reinforces an inability discourse, likely hampering recognition as well as constraining the assessment of possibility. Findings also point to institutional efforts that could make disability more legible as an expected and valued way of being in medical school. Schools can highlight that many medical students use accommodations each year and clearly define disability and possible accommodations in orientations, policies, and school handbooks (Meeks et al., 2020a). But given the power of the inability discourse, bolder efforts seem necessary to embed alternative ways of knowing disability. Incorporating critical disability studies theories into all aspects of medical education could improve disabled learners’ experiences. Such efforts could also help shift negative attitudes towards disability and access that entrench health inequities for disabled people (Lagu et al., 2022). Case studies that unearth the interpersonal dynamics of successful inclusion experiences and demonstrate systemic efforts to counteract exclusionary disability discourses would provide models for culture change and advance collective understandings of inclusive possibility.

This conceptualization of legibility supports calls for disability disclosure and accommodation decision-making structures that are led by people skilled in disability inclusion for health professions education and take up a nuanced view of disability (Bulk et al., 2019; Meeks et al., 2019b, 2021). The factors Meeks et al. (2021) specify as necessary for an informed disability disclosure structure (i.e., an arbiter of accommodation decision-making who knows medical education curriculum and assessment requirements, is conversant with best practices for accommodation and disability law) align with legibility conditions that advanced student access. However, even when such conditions were present, learners with less readily-apparent disability experiences were underserved and those with apparent disabilities still navigated stigma. Furthermore, even those school officials well-versed in disability law and accommodations for medical education periodically demonstrated views that reified the medical model of disability rather than a social or political-relational understanding of disability. Perhaps this is because the accommodation approach enshrined in disability law insufficiently challenges normalcy, focused instead on getting access to society as it currently exists (Withers, 2012; Skyer, 2019). This narrow focus ensures that educational structures need not shift, inclusion is abridged, and realist conceptions of disability are not dismantled (Donoghue, 2003). Learners are treated with skepticism and access is framed as a scarce resource, creating an antagonistic approach to inclusion (Clarke, 2022). Efforts to expand how school officials understand disability—through, for example, concepts like ableism—appear necessary to move practice beyond compliance or even “spirit of the law” approaches to disability inclusion (Jain, 2020a; Doebrich et al., 2020). Such a shift in knowing disability might ignite movement towards a more fundamental transformation of medical education that embeds disability as both valuable difference and normal human variation.

The concept of legibility was developed via data from a specific context, four U.S. medical schools that showed promise to exceed a strict compliance approach to disability inclusion, and particular participants (46 medical school administrators, faculty, and disabled medical students). The study advances insight about how individual, collective, and institutional conceptualizations of disability impact disability inclusion. However, this analysis concerned a particular context and particular individuals within it. Having followed a CGT methodology, I sought conceptual generalizability, achieving a higher-order abstraction of concepts with relevance across participants (Varpio et al., 2021). Nonetheless, the relevance of these findings and conclusions for other contexts must be assessed with attention to the particularities of a U.S. context. I have discussed legibility in relation to research from outside the US (Canada and the UK) and research that has explored disability inclusion in health professions beyond medicine. However, further research is warranted to consider the relevance of legibility and its qualities in other national contexts and health professions education disciplines. Additionally, an exploration of how to embed anti-ableism in medical education and its effects would be a valuable avenue for future research.

**Notes**.

1. I use identity-first, or disabled-persons language in this article in allegiance with the social and political-relational models of disability that contend disability is socially produced rather than a fact of the body or something someone owns.
2. I use the linguistic construction *bodymind* (rather than “body and mind” or alternating between the two) to recognize the imbrication of mind and body in resistance to Cartesian dualism (Price, 2015). In the spirit of crip politics, bodymind aims to “bring mind more centrally into debates” (Price, 2015, p. 271) about ableism, disability, and inclusion.

### Electronic supplementary material

Below is the link to the electronic supplementary material.

Supplementary Material 1

Supplementary Material 2

Supplementary Material 3

## Data Availability

Data are not publicly available due to the potential for participants to be identified.
