# Supplementary material for: Legibility: knowing disability in medical education inclusion
Source: Adv Health Sci Educ Theory Pract. 2023 Jul 21;29(2):507–30. doi: 10.1007/s10459-023-10268-1 (PMC11078834; doi:10.1007/s10459-023-10268-1)
Supplement: Supplementary file 1 — Supplementary Material 1 [file 10459_2023_10268_MOESM1_ESM.docx]

**Appendix A. Semi-structured Interview Guide – Students**

**Background**

1. What led you to pursue medical training?

2. How did you decide which medical schools to apply to?

3. What influenced your decision to attend this medical school?

**Access and Accommodations**

1. Tell me about your experiences as a medical student with a disability so far. Are there events that stick out in your mind?

2. Can you describe your experiences accessing accommodations in medical school?

3. Are there other aspects of the university experience that stand out?

4. How have you navigated disability-related barriers you have experienced in medical school?

**Disclosure**

1. Can you tell me about how you navigate disclosing your disability in medical school?

**Campus Climate**

1. How would you characterize the climate of your medical school towards students with disabilities?

2. Please tell me about your sense of belonging at medical school.

3. Can you describe a safe space where you feel you can be “out” about your disability, fully express all aspects of your identity?

4. Can you tell me about how disability is discussed in your classes and in the clinic?

**Supports and Challenges**

1. Who has been most helpful to you while in medical school? What have they been helpful with? How have they been helpful?

2. What other supportive factors have been present? How have they been supportive?

3. What have you found to be most challenging about being a medical student with a disability? How have you navigated this challenge?

**Closing Questions**

1. In a perfect world, what would positively impact your experience as a student with disability in medical school?

2. What do you think are the most important factors/tools/strategies in navigating medical school as a disabled student? How did you discover them?

3. After having these experiences, what advice would you give a person with disability interested in going to medical school?

4. Is there something else I should know to understand your experience as a medical student with a disability better?

5. Is there a question you wish I had asked?

6. Is there anything you would like to ask me?
