# Supplementary material for: Legibility: knowing disability in medical education inclusion
Source: Adv Health Sci Educ Theory Pract. 2023 Jul 21;29(2):507–30. doi: 10.1007/s10459-023-10268-1 (PMC11078834; doi:10.1007/s10459-023-10268-1)
Supplement: Supplementary file 2 — Supplementary Material 2 [file 10459_2023_10268_MOESM2_ESM.docx]

**Appendix B: Semi-structured interview guide – Faculty and Administrators**

**Background**

1. Can you tell me about your experiences at the medical school working with students with disabilities?

2. What role do you have in supporting medical students with disabilities?

3. What concerns do you have regarding students with disabilities in medicine?

**Access and Accommodations**

1. In thinking about students with disabilities, what role do you have in creating an accessible medical school environment for this community?

2. What policies/procedures regarding disability and accommodations work well? What doesn’t?

3. What support have you received from the school in understanding your obligations to students with disabilities?

**Challenges/Successes**

1. What challenges do you see in the way your medical school currently operates with regard to students with disabilities?

2. What challenges do you experience working with students with disabilities?

3. What concerns do you hear from students with disabilities?

4. What do you think your medical school does very well in creating an accessible medical school environment?

5. What do you think your medical school does very well in supporting medical school students with disabilities?

**Campus Climate**

1. How would you characterize the climate of your medical school towards students with disabilities?

**Closing Questions**

1. In a perfect world, what would you do to make medical schools more accessible to students with disabilities?

2. What do you think are the most important ways faculty/administrators can create welcoming medical school environments for students with disabilities? How did you discover these techniques?

3. Is there something else about your experiences you want me to know?

4. Is there a question you wish I had asked?

5. Is there anything you would like to ask me?
