# Supplementary material for: Legibility: knowing disability in medical education inclusion
Source: Adv Health Sci Educ Theory Pract. 2023 Jul 21;29(2):507–30. doi: 10.1007/s10459-023-10268-1 (PMC11078834; doi:10.1007/s10459-023-10268-1)
Supplement: Supplementary file 3 — Supplementary Material 3 [file 10459_2023_10268_MOESM3_ESM.docx]

**Appendix C: Semi-structured Interview Guide - Disability Service Providers**

**Role**

1. Can you tell me about your role at the university?

2. How do you conceptualize your role as a Disability Service Provider (DSP) within the larger medical school community?

**Accessibility and Accommodations**

1. Tell me about the work you do to create an accessible medical school environment for students with disabilities

2. Can you tell me how you go about determining accommodations for students at the medical school

3. What initiatives to improve access, beyond accommodations, are in place at your medical school?

**Policies and Procedures**

1. Can you share how you go about developing policies and procedures for disability services?
2. How are you involved in medical school policy creation or revision?

**Challenges and Successes**

1. How do you navigate the challenges you face in your role as a DSP?

2. What kinds of concerns do you hear from the students you work with?

3. Similarly, what concerns and challenges to you hear from faculty regarding students with disabilities?

**Campus Climate**

1. How would you characterize the climate of your medical school towards students with disabilities?

**Closing Questions**

1. In a perfect world, what would you do to improve the medical school experience for students with disabilities?

2. What do you think your medical school does very well in creating an accessible environment for students with disabilities?

3. What work have you done as a DSP that you are most proud of?

4. What do you think are the most important ways DSPs can create welcoming medical school environments for students with disabilities? How did you discover these techniques?

5. Is there something else you think I should know to understand your work as a DSP better?

6. Is there a question you wish I had asked?

7. Is there anything you would like to ask me?
